# Acceptability and confidence in antiretroviral generics of physicians and HIV-infected patients in France

**DOI:** 10.7448/IAS.17.4.19608

**Published:** 2014-11-02

**Authors:** Clotilde Allavena, Christine Jacomet, Bruno Pereira, Laurence Morand-Joubert, Haleh Bagheri, Laurent Cotte, Rodolphe Garaffo, Laurent Gerbaud, Pierre Dellamonica

**Affiliations:** 1 Service des Maladies infectieuses et tropicales, CHU Hôtel-Dieu, Nantes, France; 2 Service des Maladies infectieuses et tropicales, CHU Gabriel Montpied, Clermont-Ferrand, France; 3 Biostatistics Unit (DRCI), Clermont-Ferrand University Hospital, Clermont-Ferrand, France; 4 Virology, CHU Saint-Antoine, Paris, France; 5 Pharmacologie Médicale et Clinique, CHU Toulouse, INSERM U1027, Toulouse, France; 6 Service des Maladies infectieuses et tropicales, Hospices Civils de Lyon/INSERM U1052, Lyon, France; 7 Clinical Pharmacology, Pasteur University Hospital, Nice, France; 8 Santé Publique, CHU Clermont-Ferrand, EA 4681 PEPRADE, Clermont Université, Clermont-Ferrand, France; 9 Service des Maladies infectieuses et tropicales, CHU de l'Archet, Nice, France

## Abstract

**Introduction:**

Switching brand name medications to generics is recommended in France in the interest of cost effectiveness but patients and physicians are sometimes not convinced that switching is appropriate. Some antiretroviral (ARV) generics (ZDV, 3TC, NVP) have been marketed in France since 2013.

**Materials and Methods:**

A multicentric cross-sectional survey was performed in September 2013 to evaluate the perception of generics overall and ARV generics in physicians and HIV-infected patients and factors associated to their acceptability. Adult HIV outpatients were asked to complete a self-questionnaire on their perception of generics. Physicians completed a questionnaire on the acceptability of generics and ARV generics. Socio-demographic data, medical history and HIV history were collected.

**Results:**

116 physicians in 33 clinics (68% in University Hospital) included 556 patients (France-native 77%, active employment 59%, covered by social Insurance 100%, homosexual/bisexual contamination 47%, median HIV duration 13 years, hepatitis coinfection 16%, on ARV therapy 95%). Overall, patients accepted and had confidence in generics in 76% and 55% of the cases, respectively. Switching ARVs for generics was accepted by 44% of the patients but only by 17% if the pill burden was going to increase. 75% of the physicians would prescribe generics, but this decreased to only 26% if the combo had to be broken. The main reasons for non-prescription of generics were previous brand name ARV-induced side effects (35%), refusal of generics overall (37%), lack of understanding of generics (26%), risk of non-observance of treatment (44%), anxiety (47%) and depressive symptoms (25%). In multivariate analysis, factors associated with the acceptability of ARV generics in patients were the use of generics overall (p<0.001) and in physicians, the absence of concern regarding the drug efficacy (p<0.001) and being aware that the patient would accept generics overall (p=0.03) and ARV generics (p=0.04). No factors related to sociodemographic conditions, HIV status or comorbidities had a constrictive influence on the use of ARV generics.

**Conclusion:**

Acceptability of ARV generics in this French population is mostly dictated by the patient's and physician's knowledge and use of generics overall. Switching ARV brand name to a generic would be better accepted if the pill burden remained unchanged.

